# Recurrence and tumor-related death after resection of hepatocellular carcinoma in patients with metabolic syndrome

**DOI:** 10.1016/j.jhepr.2024.101075

**Published:** 2024-04-24

**Authors:** Giammauro Berardi, Alessandro Cucchetti, Carlo Sposito, Francesca Ratti, Martina Nebbia, Daniel M. D’Souza, Franco Pascual, Epameinondas Dogeas, Samer Tohme, Alessandro Vitale, Francesco Enrico D’Amico, Remo Alessandris, Valentina Panetta, Ilaria Simonelli, Marco Colasanti, Nadia Russolillo, Amika Moro, Guido Fiorentini, Matteo Serenari, Fernando Rotellar, Giuseppe Zimitti, Simone Famularo, Tommy Ivanics, Felipe Gaviria Donando, Daniel Hoffman, Edwin Onkendi, Yasmin Essaji, Tommaso Giuliani, Santiago Lopez Ben, Celia Caula, Gianluca Rompianesi, Asmita Chopra, Mohammed Abu Hilal, Gonzalo Sapisochin, Guido Torzilli, Carlos Corvera, Adnan Alseidi, Scott Helton, Roberto I. Troisi, Kerri Simo, Claudius Conrad, Matteo Cescon, Sean Cleary, David Choon Hyuck Kwon, Alessandro Ferrero, Giuseppe Maria Ettorre, Umberto Cillo, David Geller, Daniel Cherqui, Pablo E. Serrano, Cristina Ferrone, Luca Aldrighetti, T. Peter Kingham, Vincenzo Mazzaferro

**Affiliations:** 1 Department of Surgery, Memorial Sloan Kettering Cancer Center, New York, NY, USA; 2 Department of Surgery, San Camillo Forlanini Hospital, Rome, Italy; 3 Department of Medical and Surgical Sciences-DIME, Alma Mater Studiorum, University of Bologna, Italy; 4 Department of General and Oncologic Surgery, Morgagni-Pierantoni Hospital, Ausl Romagna, Forlì, Italy; 5 Department of Oncology and Hemato-Oncology, University of Milan, Milan, Italy; 6 Department of Surgery, HPB Surgery and Liver Transplantation, Istituto Nazionale Tumori IRCCS, Milan, Italy; 7 Hepatobiliary Surgery Division, San Raffaele Hospital Department of Surgery, Milan, Italy; 8 Department of Surgery, Massachusetts General Hospital, Boston, MA, USA; 9 Department of Surgery, McMaster University, Hamilton, ONT, Canada; 10 Department of Surgery, Paul Brousse Hospital, Villejuif, Paris, France; 11 Department of Surgery, University of Pittsburgh Medical Center, Pittsburgh, PA, USA; 12 Department of Surgical Oncological and Gastroenterological Sciences (DiSCOG), University of Padova, Padua, Italy; 13 Laltrastatistica Consultancy and Training, Biostatistics Department, Rome, Italy; 14 Department of Surgery, Mauriziano Hospital, Turin, Italy; 15 Department of Surgery, Cleveland Clinic, Cleveland, OH, USA; 16 Department of Surgery, Mayo Clinic, Rochester, MN, USA; 17 Hepato-biliary and Transplant Unit, IRCCS Azienda Ospedaliero-Universitaria di Bologna, Bologna, Italy; 18 Department of Surgery, Universidad de Navarra, Pamplona, Spain; 19 Department of Surgery, Poliambulanza Foundation Hospital, Brescia, Italy; 20 Department of General Surgery, Humanitas University and Research Hospital, IRCCS, Milan, Italy; 21 Department of Surgery, University of Toronto, Toronto, ON, Canada; 22 Department of Surgery, University of California San Francisco, San Francisco, CA, USA; 23 Department of Surgery, Texas Tech University Health Sciences Center, Lubbock, TX, USA; 24 Department of Surgery, Virginia Mason Hospital, Seattle, WA, USA; 25 Department of Surgery, Seattle Medical Center, Seattle, WA, USA; 26 Department of Surgery, Hospital Universitari Dr Josep Trueta de Girona, Girona, Spain; 27 Department of Clinical Medicine and Surgery, Università Federico Secondo, Naples, Italy; 28 Department of Surgery, Hospital, Toledo, OH, USA; 29 Department of Surgery, Saint Elizabeth Medical Center, Boston, MA, USA

**Keywords:** Hepatocellular carcinoma, Metabolic syndrome, Nonalcoholic fatty liver disease, Metabolic-associated fatty liver disease, Recurrence, Steatosis, Obesity

## Abstract

**Background & Aims:**

Metabolic syndrome (MS) is a growing epidemic and a risk factor for the development of hepatocellular carcinoma (HCC). This study investigated the long-term outcomes of liver resection (LR) for HCC in patients with MS. Rates, timing, patterns, and treatment of recurrences were investigated, and cancer-specific survivals were assessed.

**Methods:**

Between 2001 and 2021, data from 24 clinical centers were collected. Overall survival (OS), recurrence-free survival (RFS), and cancer-specific survival were analyzed as well as recurrence patterns and treatment. The analysis was conducted using a competing-risk framework. The trajectory of the risk of recurrence over time was applied to a competing risk analysis. For post-recurrence survival, death resulting from tumor progression was the primary endpoint, whereas deaths with recurrence relating to other causes were considered as competing events.

**Results:**

In total, 813 patients were included in the study. Median OS was 81.4 months (range 28.1–157.0 months), and recurrence occurred in 48.3% of patients, with a median RFS of 39.8 months (range 15.7–174.7 months). Cause-specific hazard of recurrence showed a first peak 6 months (0.027), and a second peak 24 months (0.021) after surgery. The later the recurrence, the higher the chance of receiving curative intent approaches (*p* = 0.001). Size >5 cm, multiple tumors, microvascular invasion, and cirrhosis were independent predictors of recurrence showing a cause-specific hazard over time. RFS was associated with death for recurrence (hazard ratio: 0.985, 95% CI: 0.977–0.995; *p* = 0.002).

**Conclusions:**

Patients with MS undergoing LR for HCC have good long-term survival. Recurrence occurs in 48% of patients with a double-peak incidence and time-specific hazards depending on tumor-related factors and underlying disease. The timing of recurrence significantly impacts survival. Surveillance after resection should be adjusted over time depending on risk factors.

**Impact and implications:**

Metabolic syndrome (MS) is a growing epidemic and a significant risk factor for the development of hepatocellular carcinoma (HCC). The present study demonstrated that patients who undergo surgical resection for HCC on MS have a good long-term survival and that recurrence occurs in almost half of the cases with a double peak incidence and time-specific hazards depending on tumor-related factors and underlying liver disease. Also, the timing of recurrence significantly impacts survival. Clinicians should therefore adjust follow-up after surgery accordingly, considering timing of recurrence and specific risk factors. Also, the results of the present study might help design future trials on the use of adjuvant therapy following resection.

## Highlights

• Patients with MS undergoing LR for MS have good long-term survival.
• Recurrence occurs in 48% of patients with a double-peak incidence.
• Time-specific hazard of recurrence depends on tumor-related factors and underlying liver disease.
• The timing of recurrence significantly impacts survival.

## Graphical abstract

**Figure:**
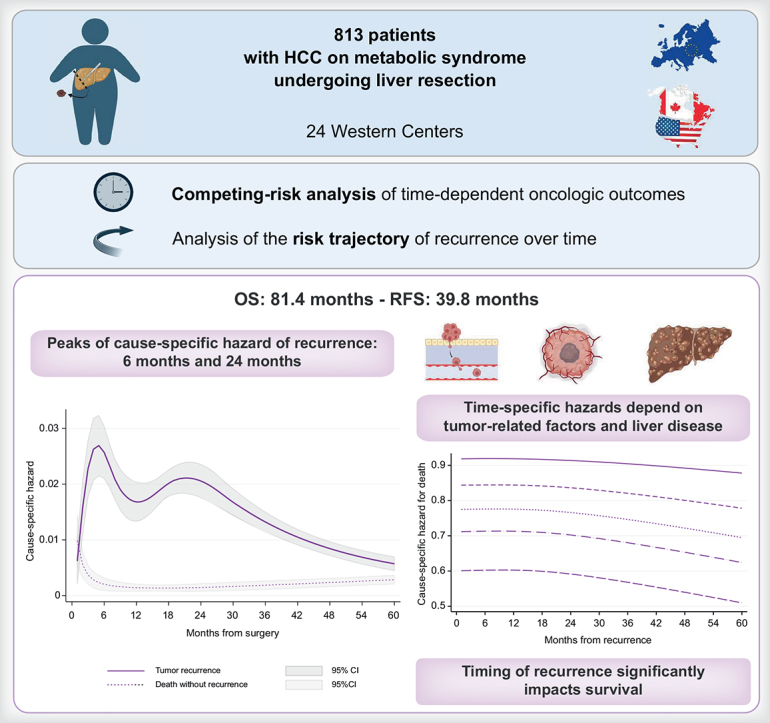

## Introduction

Metabolic syndrome (MS) includes a cluster of inter-related clinical conditions and is currently considered a disease of epidemic proportions in the high-income countries.^1^ Nonalcoholic fatty liver disease (NAFLD) is the hepatic manifestation of MS. Liver steatosis is the initial common histopathological modification associated with NAFLD. In most cases, patients remain free of inflammation; however, 10–20% of patients who have fatty liver will develop nonalcoholic steatohepatitis, fibrosis, cirrhosis, and eventually hepatocellular carcinoma (HCC).^2^^,^^3^ The incidence of HCC in patients with MS is rising and is projected to increase worldwide further.^4^ Management of these patients is complex. Liver resection (LR) remains a valuable and potentially curative treatment option, but morbidity and mortality are high.[5], [6], [7]

Substantial clinical and oncological differences exist between patients with HCC on MS and those with other underlying etiologies, such as viral or alcoholic diseases. Indeed, it has been estimated that 30–60% of patients with MS develop HCC without underlying fibrosis or cirrhosis.^6^^,^^8^^,^^9^ Furthermore, distinctive environmental factors, endocrine dysregulations, and specific genetic alterations have been linked to hepatocarcinogenesis in the absence of cirrhosis.^8^^,^[10], [11], [12] Whether these peculiar features are associated with distinct oncological outcomes is under investigation. Previous retrospective studies showed that patients with MS undergoing LR for HCC have excellent survival and improved long-term outcomes compared with patients with viral or alcoholic liver disease.^6^^,^^7^^,^[13], [14], [15] By contrast, other authors reported opposite results and worse survival figures.^16^^,^^17^ Nevertheless, tumor recurrence following hepatectomy remains high, and information regarding prognostic factors associated with relapse and oncological outcomes is lacking. Survival depends on the complex interaction between a patient’s age, comorbidities, tumor burden, and the status of the nontumoral liver parenchyma. Furthermore, the impact of tumor recurrence on the long-term survival of patients with HCC and MS remains unknown. In this setting, understanding the prognostic impact of clinicopathological characteristics and nontumoral parenchymal changes on recurrence and survival is of utmost importance.

In the present study, the long-term outcomes of LR for HCC in patients from high-income countries with MS were collected from a large multicenter database. Rates, timing, patterns, and treatment of recurrences were investigated, and cancer-specific survivals were assessed.

## Methods

Between January 2001 and January 2021 (based on the date of LR), data from 24 centers (12 European and 12 North American) were collected. Patients were included only if fulfilling the following criteria: (i) undergoing LR for histologically proven HCC; (ii) a preoperative diagnosis of MS, defined by three out of five of the following criteria^18^: (a) abdominal obesity [body mass index (BMI) ≥30 kg/m^2^ or waist circumference >102 cm in men and >88 cm in women]; (b) triglycerides >150 mg/dl; (c) high-density lipoprotein cholesterol <40 mg/dl in men and <50 mg/dl in women; (d) type 2 diabetes mellitus or glucose intolerance (fasting glucose >110 mg/dl); or (e) hypertension (blood pressure >130/85 mmHg); and (iii) older than 18 years of age. The following exclusion criteria were applied: (i) resections of HCC on viral, alcoholic (>40 g/d, >21 drinks per week for men and >14 drinks per week for women), or autoimmune diseases, as well as hemochromatosis and Wilson’s disease; (ii) fibrolamellar HCC or mixed hepatocellular-cholangiocellular carcinoma; (iii) extrahepatic metastases; (iv) exploratory laparoscopy/laparotomy without LR; (5) main portal vein, hepatic artery, biliary duct, or inferior vena cava invasion. The primary endpoint was to investigate the oncological outcomes focusing on recurrence rates, timing, patterns, and treatments, as well as cancer-specific survivals. Predictive factors of tumor relapse over time were explored. Institutional Review Board (IRB) approval was obtained from the coordinating center (no. 16-801, approved December 7, 2020); data transfer agreement and IRB approval were requested for all participating institutions. Each case was discussed in a multidisciplinary setting, and informed consent for surgery was obtained from each patient. Major LR was defined as the resection of three or more segments. Morbidity was graded according to the Clavien–Dindo classification.^19^ A surgical margin of <1 mm was considered an R1 resection. Pathological nontumoral liver tissue information was collected: degree of fibrosis, steatosis, lobular inflammation, and hepatocellular ballooning were graded according to the NAFLD Activity Score (NAS).^20^ Resection, ablation, or liver transplantation were considered curative intent treatments of recurrence. Trans-arterial radio- or chemoembolization was defined as a locoregional approach.

### Statistical analysis

Continuous data were expressed as median and IGR (25th and 75th). Categorical data were expressed as numbers and percentages. Trends over time were evaluated with the Cochran–Armitage test. The median length of follow-up was estimated using the reverse Kaplan–Meier estimator. Recurrence-free survival (RFS) was the primary endpoint, and was estimated from surgery until evidence of tumor relapse. Deaths occurring without evidence of tumor recurrence were considered competing events. Patients not experiencing the event were censored at the final follow-up. Thus, the entire survival analysis was conducted according to a competing-risk framework. The analysis aimed to delineate the trajectory of the specific risk of tumor recurrence over time; therefore, a time-dependent approach was applied to the competing risk analysis. When evaluating post-recurrence survival, death resulting from tumor progression was the primary endpoint, whereas deaths with recurrence resulting from other causes were considered competing events. Patients not experiencing these events were censored at the final follow-up. All the analyses were conducted using the Stata module ‘stpm2cr’ (Stata Corporation, College Station, TX, USA), which is a flexible parametric module able to model all cause-specific incidence functions simultaneously and covariate effects on all competing causes. Four degrees of freedom (df) were applied for fixed covariates and three df for time-dependent variables. Results were reported as cause-specific hazards (CSH) or CSH ratios together with 95% CIs.

## Results

In total, 1,100 patients were gathered within the study period. Of these, 287 were excluded because of missing clinical or pathological data. The final study population comprised 813 individuals [median 20 (IQR 7–42) cases per center] with complete clinicopathological data (Fig. S1). All patients underwent surgical treatment of a newly diagnosed HCC without preoperative treatments (Table 1). Of note, the median tumor size was 5 cm, a microscopic R0 resection margin was observed in 91.9% of patients, and macrovascular invasion was diagnosed in 17.2% of patients. Background liver parenchyma was normal or had minimal fibrosis (F0 or F1) in 344 patients (42.3%), whereas 198 showed cirrhosis (24.4%). The surgical morbidity was 32.5% (12.8% major complications), with a 90-day mortality of 2.8%.

**Table 1:** Clinicopathological characteristics of the study population.

Variable	N = 813∗
**Clinical features**	
Age (years)	69 (63–75)
Male	570 (70.1)
Inclusion period of time	
2001–2007	100 (12.4)
2008–2014	285 (35.1)
2015–2021	428 (52.6)
Geographic area	
Europe	405 (49.8)
North America	408 (50.2)
ASA score III to IV	516 (63.5)
BMI (kg/m^2^)	29.2 (25.4–32.5)
Obesity (BMI ≥30 kg/m^2^)	349 (42.9)
Hypertension	631 (77.6)
Diabetes	462 (56.8)
Ischemic heart disease	170 (21.0)
Respiratory disease	128 (15.7)
**Surgical characteristics**	
Minimally invasive approach	339 (41.7)
Type of resection	
Limited resection	215 (26.5)
Segmentectomy	140 (17.2)
Sectionectomy	208 (25.6)
Hemi-hepatectomy	212 (26.1)
Trisectionectomy	38 (4.7)
Major hepatectomy	250 (30.8)
**Histological characteristics**	
Nontumoral liver fibrosis	
F0 or F1	344 (42.3)
F2	107 (13.2)
F3	164 (20.2)
F4	198 (24.4)
Degree of steatosis	
<5%	285 (35.1)
5–33%	314 (38.6)
>33%	214 (26.3)
Number of lesions	
Single	688 (84.6)
Two or three	98 (12.1)
More than three	27 (3.3)
Size of lesions (cm)	5.0 (3.2–7.5)
R0 resection	747 (91.9)
G3/G4 tumor grade	139 (17.2)
Macrovascular invasion	139 (17.2)
Microvascular invasion	354 (43.5)
Satellitosis	149 (18.3)
**Follow-up events**	
Recurrence	392 (48.2)
Recurrence-free survival (months)	39.8 (15.7–174.7)
Recurrence site	
Intrahepatic	284/392 (72.4)
Intra- and extrahepatic	49/392 (12.5)
Extrahepatic	37/392 (9.4)
Not described	22/392 (5.6)
Treatment of recurrence	
Potentially curative (resection, transplantation, ablation)	124/392 (31.6)
Locoregional (TACE/TARE, radiation)	107/392 (27.3)
Systemic treatments	39 (10.0)
Best supportive care/none	27 (6.9)
Unknown	95 (24.2)
Death	
Tumor-related death	189 (23.3)
Liver-related death	42 (5.2)
Cardiopulmonary event	37 (4.6)
Other	44 (5.4)
Overall survival (months)	81.4 (28.1–157.0)

∗ Data are presented as mean (range), n (%), or n/N (%).

During follow-up, 312 patients died (38.3%; median follow-up 53 months; IQR: 25–89). Median overall survival of the entire cohort was 81.4 months (IQR: 28.1–157.0). Of the patients, 393 patients developed tumor recurrence (48.3%), with a median RFS of 39.8 (IQR: 15.7–174.7). In total, 81 patients (10.0%) died from causes other than tumor progression. Of these, 32 died because of liver failure (39.5%), 25 from cardiovascular or pulmonary events (30.9%), and 24 from other causes (29.6%).

### Time-specific hazards of recurrence, recurrence patterns, and treatments

**Fig. 1:**
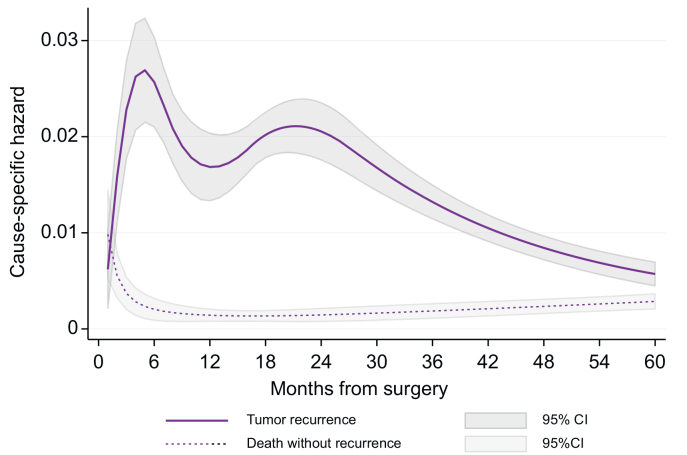
Time-specific hazards of hepatocellular carcinoma (HCC) recurrence and death without tumor recurrence.

The time course of recurrence-specific risk is depicted in Fig. 1. A first peak was observed 6 months after surgery (CSH peak: 0.027), followed by a decrease, with the recurrence risk reaching a nadir at 12 months (CSH: 0.017). Subsequently, a second peak in recurrence-specific risk was observed ∼24 months after resection (CSH: 0.021), before progressively decreasing thereafter. Conversely, the time course of mortality without evidence of tumor recurrence showed a first expected peak early after surgery (CSH: 0.0098), which promptly dropped within the first year (CSH: 0.0014) and progressively increased after 24 months.

**Fig. 2:**
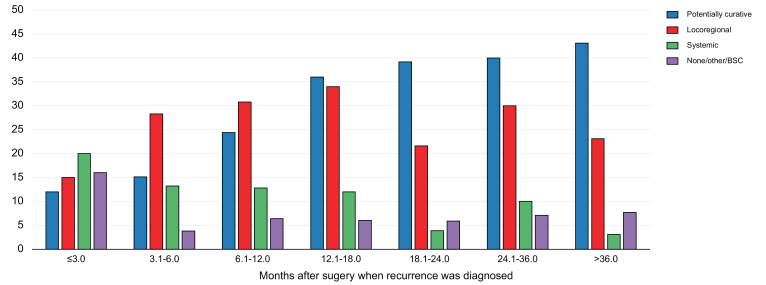
Treatment of recurrences over time. Potentially curative: resection, ablation, transplantation; Locoregional: radioembolization or chemoembolization. BSC, best supportive care.

Of the 393 patients experiencing tumor relapse (48.3%), 284 had recurrence in the liver as the only site (72.4%), whereas 86 had systemic recurrence with or without involvement of the liver (21.9%). In terms of the treatment of recurrences (Fig. 2), 124 patients (31.6%) were treated by potentially curative approaches (76 repeated resections, 44 ablations, and four liver transplantations), 107 underwent locoregional treatments (27.3%), and 66 received systemic therapy or best supportive care (16.9%). Potentially curative treatments were adopted in 29.7% of patients with F0 or F1 fibrosis, in 32.6% of patients with F2 fibrosis, in 35.2% of patients with F3 fibrosis, and in 31.8% of patients with F4 fibrosis (*p* = 0.86). The later the recurrence, the higher the proportion of patients who received curative intent approaches *(p* = 0.001), and the lower the proportion of patients who received systemic therapy (*p* = 0.017). Conversely, locoregional treatments in the form of radio- and chemoembolization remained steady over time (*p* = 0.884).

### Predictors of recurrence and death without recurrence

In terms of clinicopathological features, the following variables predicted HCC recurrence during follow-up one univariate analysis with different hazards over time: tumor grade III or IV, size >5 cm, macrovascular invasion, R1 resection, microvascular invasion, multiple tumors, satellites, and cirrhosis (Table 2). After multivariable analysis (Table 3), size >5 cm, multiple tumors, microvascular invasion, and cirrhosis independently showed a CSH over time.

**Table 2:** Univariate analysis of time-specific hazard ratios for predictors of hepatocellular carcinoma recurrence and death without tumor recurrence.

Event/characteristic	Months since hepatic resection						
	3	6	12	18	24	36	60
**Tumor recurrence**							
Diameter >5 cm	2.52 (1.31–3.73)∗	1.80 (1.05–2.55)∗	1.24 (0.84–1.64)	1.23 (0.85–1.61)	1.17 (0.86–1.48)	1.01 (0.63–1.36)	0.86 (0.27–1.44)
Macrovascular invasion	2.23 (1.15–3.30)∗	2.02 (1.37–2.65)∗	1.66 (1.05–2.27)∗	1.54 (0.98–2.10)	1.33 (0.89–1.78)	0.77 (0.35–1.20)	0.56 (0.07–1.06)
Microvascular invasion	2.71 (1.45–3.98)∗	3.08 (1.80–4.36)∗	2.01 (1.37–2.64)∗	1.70 (1.19–2.21)∗	1.46 (1.08–1.84)∗	0.91 (0.56–1.27)	0.57 (0.11–1.02)
Satellitosis	2.66 (1.42–3.91)∗	2.27 (1.27–3.27)∗	1.82 (1.12–2.53)∗	1.75 (1.13–2.38)∗	1.61 (1.11–2.11)∗	1.18 (0.52–1.83)	0.82 (0.01–1.84)
Grade III or IV	1.88 (1.06–2.69)∗	1.47 (1.02–1.92)∗	1.17 (0.80–1.55)	1.17 (0.89–1.46)	1.11 (0.85–1.38)	0.92 (0.53–1.33)	0.76 (0.18–1.34)
Parenchymal R1	2.44 (1.07–3.80)∗	1.48 (0.73–2.23)	1.16 (0.44–1.87)	1.45 (0.96–1.94)	1.67 (0.95–2.41)	1.82 (0.77–2.87)	2.27 (0.19–4.33)
Multiple tumors	2.35 (1.18–3.51)∗	2.36 (1.29–3.42)∗	2.16 (1.36–2.96)∗	2.02 (1.30–2.74)∗	1.84 (1.26–2.43)∗	1.34 (0.65–2.02)	0.91 (0.01–1.95)
Cirrhosis	1.36 (0.66–2.05)	1.75 (0.97–2.52)	1.33 (0.83–1.84)	1.36 (0.90–1.83)	1.64 (1.19–2.09)∗	2.80 (1.78–3.83)∗	5.57 (1.72–9.41)∗
**Death without recurrence**							
Minimally invasive	0.58 (0.29–0.87)∗	0.74 (0.45–1.03)	0.80 (0.58–1.03)	0.94 (0.68–1.19)	0.85 (0.61–1.09)	0.94 (0.68–1.19)	1.21 (0.47–1.96)
Major hepatectomy	2.29 (1.06–3.52)∗	1.68 (1.10–2.27)∗	1.10 (0.71–1.48)	0.91 (0.65–1.15)	0.84 (0.49–1.18)	0.80 (0.38–1.23)	0.77 (0.27–1.26)
Cirrhosis	1.73 (0.75–2.71)	1.56 (0.96–2.16)	1.32 (0.84–1.79)	1.51 (1.06–1.97)∗	1.85 (1.38–2.33)∗	2.66 (1.68–3.63)∗	4.19 (1.74–6.64)∗

For simplicity, among the variables reported in Table 1 in the main text, only variables showing a cause-specific hazard ratio with *p* <0.05 at some time-point were reported. Consequently, those not reported did not show a relationship with the specific endpoint at any time point considered (Tables S1 and S2).

∗ *p* <0.05.

**Table 3:** Multivariate analysis of time-specific hazard ratios for predictors of hepatocellular carcinoma recurrence and death without tumor recurrence.

Event/characteristic	Months since hepatic resection						
	3	6	12	18	24	36	60
**Tumor recurrence**							
Diameter >5 cm	2.54 (1.31–3.76)∗	1.81 (1.04–2.57)∗	1.24 (0.83–1.64)	1.21 (0.83–1.64)	1.13 (0.84–1.43)	0.94–0.61–1.27)	0.77 (0.27–1.29)
Microvascular invasion	2.53 (1.31–3.74)∗	2.93 (1.66–4.19)∗	2.02 (1.35–2.70)∗	1.72 (1.17–2.26)∗	1.47 (1.06–1.89)∗	0.94 (0.56–1.32)	0.56 (0.08–1.04)
Multiple tumors	1.86 (0.94–2.77)	1.92 (1.27–2.56)∗	1.72 (1.10–2.33)∗	1.57 (1.13–2.02)∗	1.42 (1.02–1.83)∗	1.05 (0.51–1.59)	0.74 (0.02–1.45)
Cirrhosis	1.41 (0.68–2.14)	1.80 (0.99–2.61)	1.42 (0.88–1.96)	1.46 (0.96–1.96)	1.73 (1.24–2.21)∗	2.87 (1.84–4.61)∗	5.25 (1.66–8.84)∗
**Death without recurrence**							
Minimally invasive	0.59 (0.29–0.88)∗	0.75 (0.46–1.04)	0.81 (0.58–1.04)	0.83 (0.59–1.07)	0.86 (0.62–1.12)	0.97 (0.70–1.23)	1.25 (0.49–2.02)
Major hepatectomy	2.20 (1.16–3.23)∗	1.74 (1.09–2.40)∗	1.17 (0.82–1.52)	1.04 (0.73–1.36)	0.99 (0.69–1.29)	0.99 (0.72–1.27)	1.03 (0.39–1.66)
Cirrhosis	1.56 (0.75–2.37)	1.74 (1.03–2.45)∗	1.58 (1.10–2.07)∗	1.59 (1.09–2.09)∗	1.70 (1.19–2.21)∗	2.19 (1.60–2.78)∗	4.09 (1.70–6.49)∗

∗ *p* <0.05.

**Fig. 3:**
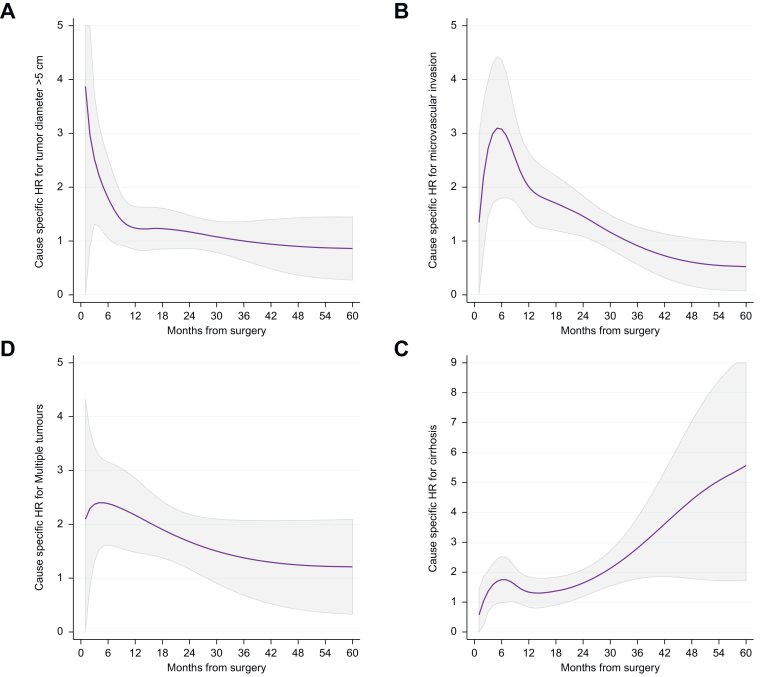
Time-specific HRs of tumor size >5 cm, microvascular invasion, multiple tumors, and cirrhosis for HCC recurrence. Gray areas represent 95% CIs; consequently, the lower limit above 1 indicates *p* <0.05. HCC, hepatocellular carcinoma.

In detail (Fig. 3), size >5 cm showed an impact on the cause-specific hazard for recurrence between the second and sixth month after surgery (*p* <0.05) and was not significant thereafter; microvascular invasion increased the hazard between the second and 26th month after surgery (*p* <0.05) and was not significant thereafter. Similarly, the presence of multiple tumors increased the hazard between the third and 24th month after surgery (*p* <0.05) and was not significant thereafter. Conversely, cirrhosis was not associated with recurrence earlier during follow-up, but increased the hazard from the 20th month onward (*p* <0.05).

Only major hepatectomy, type of surgical approach, and cirrhosis were associated with increased hazard for death without recurrence at univariate analysis (Table 2). After multivariable analysis (Table 3), major hepatectomy increased the probability of death within the first 6 months from surgery (*p* <0.05), then the hazard ratio (HR) progressively decreased, whereas the minimally invasive approach decreased the risk within the first 3 months. Conversely, the presence of cirrhosis increased the HR starting from 6 months after surgery (*p* <0.05).

### Mortality after recurrence

**Fig. 4:**
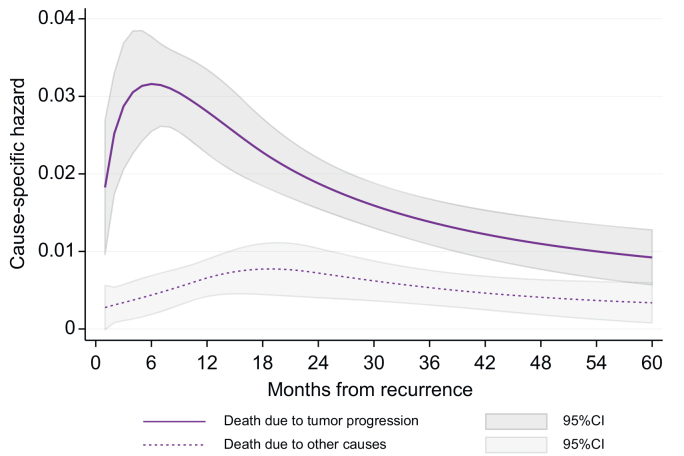
Time-specific hazards of death resulting from tumor progression and death from causes other than progression from the date of tumor relapse.

**Fig. 5:**
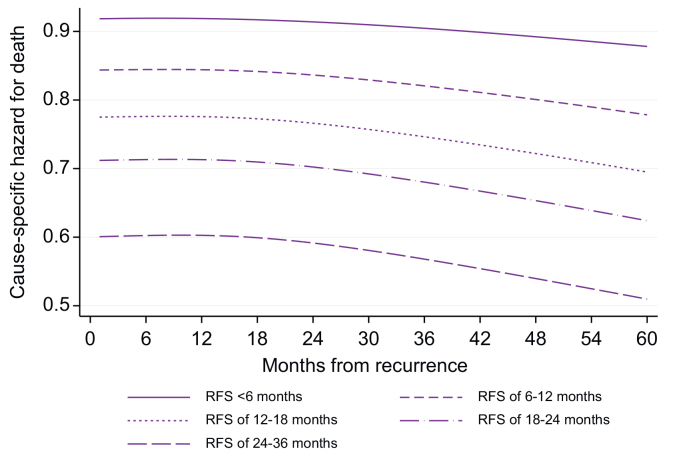
Time-specific hazards of death resulting from tumor recurrence according to RFS. RFS, recurrence-free survival.

The time-specific hazard of death for tumor progression peaked 6 months after the diagnosis of recurrence (CSH: 0.032), followed by a progressive decrease (Fig. 4). Conversely, the time-specific hazard of dying from causes other than tumor progression remained stable over time, with a slightly higher risk within 2 years from the diagnosis of recurrence (CSH: 0.008). The time to recurrence was associated with death for tumor recurrence (HR 0.985; 95% CI: 0.977–0.995; *p* = 0.002) meaning that the earlier the recurrence, the higher the chance of dying from tumor progression (Fig. 5). Conversely, RFS was not significantly associated with death due to causes other than tumor progression (HR: 1.007; 95% CI: 0.994–1.020; *p* = 0.319).

## Discussion

In the present study, we observed that patients with MS undergoing LR for HCC had good long-term survival. However, outcomes were worsened by tumor recurrence, which occurred in approximately half of cases. On the one hand, tumor-related factors were associated with recurrence early after surgery, which was treated predominantly with systemic therapy, hence leading to worse survival. On the other hand, patients with cirrhosis developed disease relapse later in follow-up, being potentially curable and, thus, displaying a better prognosis.

MS is a growing healthcare issue, and NAFLD currently represents the most common chronic liver disease in high-income countries.^21^ Patients with MS have higher long-term mortality compared the general population because they have multiple cardiovascular, metabolic, and respiratory comorbidities. Furthermore, they are more prone to develop cancer.^22^ Specifically, a 2.13-fold increased risk of developing HCC has been reported, with distinct features and specific prognosis.^23^ Indeed, even though patients with NAFLD might develop cirrhosis, a well-known risk factor for HCC, not only patients with cirrhosis and MS develop HCC. Cauchy *et al.* reported that only 30% of patients with MS develop HCC on significant fibrosis or cirrhosis and this figure is consistent with the present study, in which 44% of patients had F3 or F4 fibrosis and more than 42% had the absence of. or negligible, fibrosis.^6^ This is because hepatocarcinogenesis in this setting is not only the result of the abnormal regeneration induced by cirrhosis, but also the consequence of the proinflammatory state and the endocrine disequilibrium that exists in such patients.^8^^,^^12^^,^^24^

The screening of patients with MS at high risk for HCC remains problematic. Previous research showed that, because of the lack of surveillance, these patients present at more advanced stages, with greater tumor burden, infiltrative patterns, and higher α-fetoprotein levels.^1^ Nevertheless, when diagnosis is made at an earlier stage and curative treatments can be pursued, long-term outcomes are excellent.^1^^,^^15^ Patients with MS and treated with LR are generally younger and have better liver function compared with those with HCV- or alcohol-related disease, and a survival rate of up to 91% has been reported at 5 years.^1^^,^^25^ In a recent study, Vitale *et al.* demonstrated that patients with MS have a higher chance of dying from causes other than HCC but a lower chance of dying from cancer compared with patients with HCC from other etiologies and that their median OS is significantly better at all stages of the disease.^26^ Our study confirmed these excellent outcomes, with a median OS of >7 years. Therefore, the dismal outcomes reported by some authors should not be interpreted as a surrogate of more aggressive tumor biology, but rather as the consequence of delayed diagnosis, affecting the chance of adopting potentially curative treatments.^26^ This observation is reinforced by a large, matched study by Viganò *et al.* that accounted for confounders; these authors showed that patients with MS have better OS, fewer recurrences, and longer disease-free intervals compared with those with underlying HCV-related disease.^13^ In the same study, tumor-related factors, such as the number of nodules, satellites, and microvascular invasion, consistently affected survival.^13^ In the present cohort, we confirmed tumor histopathological characteristics as determinants of oncological outcomes, but also highlighted that these were not the only prognostic variable, with other prognostic variables coming into play with different hazards at different time points, that is, tumor-related factors predicted recurrence early after surgery; underlying liver cirrhosis was associated with relapse later in time; and extension of hepatectomy and cirrhosis itself can mask the true magnitude of this occurrence.

Different thresholds have been used in the literature to define early and late recurrences, with 2 years being the most widely adopted.^27^^,^^28^ The time-dependent approach of the present study suggests that categorizing recurrences using specific thresholds is somewhat limited. Indeed, earlier and later recurrences are not mutually exclusive, because patients with significant risk factors for early recurrences might still develop relapse later in time. Rather, this is better expressed continuously, with different risk probabilities over time. Our results showed that the hazard of recurrence has a double peak in incidence, one at ∼6 months and one at 22 months (Fig. 1). Most interestingly, these two peaks were not the same in all patients (Fig. 3); instead, the interplay of different predictors increased the hazard of recurrence, its timing, and, consequently, its treatment. Indeed, on the one hand, patients with aggressive tumor characteristics (*i.e.* large and/or multiple tumors with microvascular invasion) in the absence of cirrhosis were most likely to recur early after surgery, were not amenable to curative intent treatment strategies, and, therefore, showed poor survival. On the other hand, patients with indolent tumoral characteristics but with underlying liver cirrhosis had increased hazards of recurrence starting ∼2 years after surgery. In these cases, treatment was most likely to have a curative intent when relapse occurred, eventually displaying good survival after recurrence. Finally, patients with both cirrhosis and negative tumoral characteristics were persistently at higher risk of recurrence with different hazards over time. The above-mentioned considerations could guide the selection of candidates for hepatectomy. Patients presenting with unfavorable tumor characteristics are probably not the ideal candidates for surgery because they will likely recur early and treatment will fail. Considering the hierarchy of HCC treatment, the possibility for a test-of-time strategy adopting neoadjuvant or bridging approaches could be considered, even though this might eventually result in a loss of potentially curative options.^29^^,^^30^

Conversely, in the absence of aggressive tumor behavior, surgery represents a potentially curative strategy associated with good survival even in cirrhosis, because recurrences occur mostly later in follow-up and are treatable. Our findings might also help adjust surveillance after resection in such patients, intensifying early observation in those with aggressive tumor characteristics and prolonging follow-up in those with underlying cirrhosis.^31^ Finally, because data on adjuvant therapy are emerging, patients at high risk of early recurrence might be included in active clinical trials, whereas later relapse might be prevented halting the progression chain of parenchymal changes from steatosis to steatohepatitis and fibrosis.[32], [33], [34], [35], [36] In 2021, Kim *et al.* came to different conclusions using a similar analysis. The hazard of recurrence in their cohort peaked at 1 year after surgery, steadily decreasing thereafter to 5.3%/year after 5 years.^37^ However, this study included 84.6% of patients with HBV-related HCCs, which have peculiar characteristics and distinct long-term outcomes, justifying our different results. To the best of our knowledge, this is the first report of time-specific hazards of recurrence in a homogenous and large cohort of patients with MS. Our study provides the first evidence of the oncological outcomes following hepatectomy in such patients. Given that HCC on MS is projected to become the most prevalent liver disease worldwide, information on recurrences and survivals following curative intent treatments is key to improving patient selection and planning surveillance.

Given its retrospective design, this study has some limitations. Data from the present cohort came from surgical units and, thus, only patients undergoing surgery were enrolled and only pathological data from resected specimens were collected. Unfortunately, this is difficult to overcome because biopsies in nonsurgical patients are often unjustified. All cases were deemed resectable, whereas, in clinical practice, patients might present at later stages. Consequently, the good survival figures should not be generalized to all patients with HCC on MS. We must also acknowledge that, unfortunately, we had no data regarding cases excluded from surgery at each of the involved institutions. Comparisons between such cohorts of patients should be encouraged because they could provide interesting information on the natural history of the disease. Our study specifically excluded patients with alcoholic intake based on the number of drinks per week. Given that this is a retrospective multicenter study, some patients with alcoholic liver disease might have been included by mistake, given the variability in the data gathering at each center. These patients could be defined as affected by metabolic and alcohol-related/associated liver disease (MetALD) according to recent work.^38^ Finally, because this is a multicenter study, there could be substantial heterogeneity in the surgical practice and the interpretation of histopathological features.

In conclusion, patients with MS undergoing LR for HCC without previous treatment had good long-term survival. Recurrence occurred in 48% of patients with time-specific hazards depending on tumor-related factors and underlying liver disease. Multiple lesions, size >5 cm, and microvascular invasion increased the risk of recurrence early after surgery. Patients with cirrhosis might develop recurrence later during follow-up, and this feature masks, together with the extent of hepatectomy, the true magnitude of tumor recurrence. The timing of recurrence significantly impacts survival. Surveillance after resection should be adjusted over time depending on specific risk factors, which could become targets for new prevention strategies.

## Abbreviations

BMI, body mass index; CSH, cause-specific hazards; DF, degrees of freedom; HCC, hepatocellular carcinoma; HZ, hazard ratio; IRB, Institutional Review Board; LR, liver resection; MetALD, metabolic and alcohol-related/associated liver disease; MS, metabolic syndrome; NAFLD, nonalcoholic fatty liver disease; NAS, NAFLD activity score; OS, overall survival; RFS, recurrence-free survival.

## Financial support

The authors have no financial support to disclose.

## Conflicts of interest

The authors declare no conflicts of interest that pertain to this work.

Please refer to the accompanying ICMJE disclosure forms for further details.

## Authors’ contributions

Conceived and designed the analysis: GB, AC, CS, VM, TPK. Data collection: FR, MN, DMD, FP, ED, ST, AV, FD, RA, MC, NR, AM, GF, MS, FR, GZ, SF, TI, FGD, DH, EO, YE, TG, SLB, CC, GR, AC, MAH, GS, GT, CC, AA, SH, RIT, KS, CC, MC, SC, CHDK, AF, GME, UC, DG, DC, PES, CF, LA. Analyzed the data: GB, AC, VP, IS. Wrote the paper: GB, AC, CS, VM, TPK. Revised and approved the paper: FR, MN, DMD, FP, ED, ST, AV, FD, RA, MC,NR, AM,GF, MS, FR, GZ, SF, TI, FGD, DH, EO, YE, TG, SLB, CC, GR, AC, MAH, GS, GT, CC, AA, SH, RIT, KS, CC, MC, SC, CHDK, AF, GME, UC, DG, DC, PES, CF, LA.

## Data availability statement

The data that support the findings of the study are available on request from the corresponding author (GB).
