# Supplementary material for: Recurrence and tumor-related death after resection of hepatocellular carcinoma in patients with metabolic syndrome
Source: JHEP Rep. 2024 Apr 24;6(7):101075. doi: 10.1016/j.jhepr.2024.101075 (PMC11220535; doi:10.1016/j.jhepr.2024.101075)
Supplement: Multimedia component 1 [file mmc1.pdf]

Recurrence and tumor-related death after resection of hepatocellular carcinoma in patients with metabolic syndrome

Giammauro Berardi, Alessandro Cucchetti Carlo Sposito, Francesca Ratti, Martina Nebbia, Daniel M. D'Souza, Franco Pascual, Epameinondas Dogeas, Samer Tohme, Alessandro Vitale, Francesco D'Amico, Remo Alessandris, Valentina Panetta, Ilaria Simonelli, Marco Colasanti, Nadia Russolillo, Amika Moro, Guido Fiorentini, Matteo Serenari, Fernando Rotellar, Giuseppe Zimitti, Simone Famularo, Tommy Ivanics, Felipe Gaviria Donando, Daniel Hoffman, Edwin Onkendi, Yasmin Essaji, Tommaso Giuliani, Santiago Lopez Ben, Celia Caula, Gianluca Rompianesi, Asmita Chopra, Mohammed Abu Hilal, Gonzalo Sapisochin, Guido Torzilli, Carlos Corvera, Adnan Alseidi, Scott Helton, Roberto I. Troisi, Kerri Simo, Claudius Conrad, Matteo Cescon, Sean Cleary, Choon Hyuck David Kwon, Alessandro Ferrero, Giuseppe Maria Ettorre, Umberto Cillo, David Geller, Daniel Cherqui, Pablo E. Serrano, Cristina Ferrone, Luca Aldrighetti, T. Peter Kingham, Vincenzo Mazzaferro

Table of contents

Fig. S1……………………………………………………………………………………2
Table S1…………………………………………………………………………………3
Table S2…………………………………………………………………………………4

Fig. S1. Flowchart diagram of the collected sample.

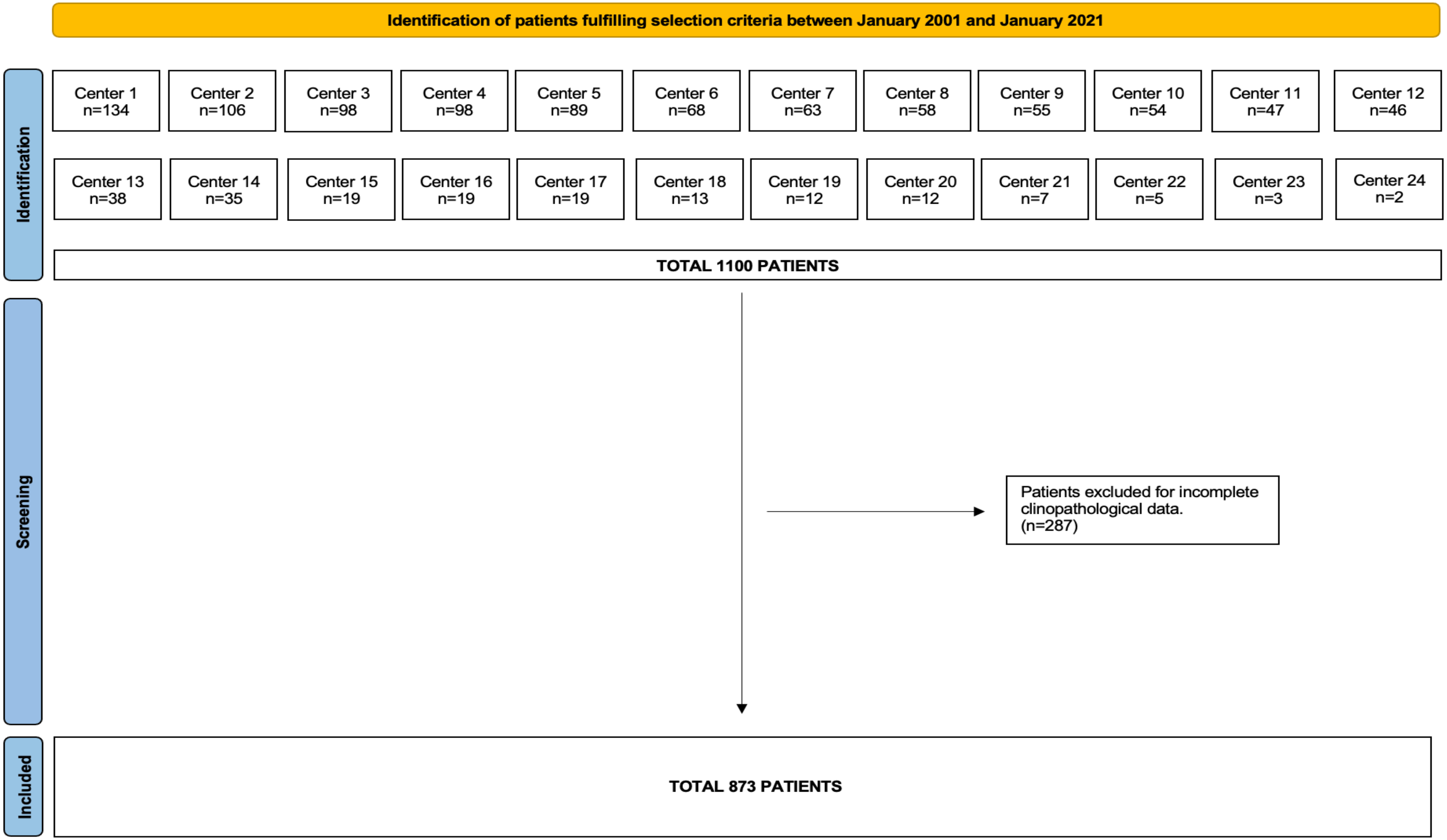

Table S1. Univariate analysis of time-specific hazard ratios for predictors of HCC recurrence.

Variable	6-m	12-m	24-m	60-m
**Clinical features**				
Age ≥70 years	0.95 (0.57, 1.34)	1.02 (0.69, 1.34)	1.06 (0.78, 1.33)	1.12 (0.37, 1.87)
Male	0.98 (0.55, 1.40)	1.08 (0.79, 1.46)	1.08 (0.78, 1.39)	1.14 (0.30, 2.01)
Inclusion period of time				
2001 – 2007	Ref.	Ref.	Ref.	Ref.
2008 – 2014	0.75 (0.48, 1.04)	0.76 (0.47, 1.04)	0.75 (0.47, 1.02)	0.71 (0.45, 1.01)
2015 – 2021	1.08 (0.83, 1.33)	1.08 (0.83, 1.33)	1.08 (0.84, 1.34)	1.09 (0.84, 1.34)
Geographic area				
Europe	Ref.	Ref.	Ref.	Ref.
North America	1.52 (0.91, 2.13)	0.89 (0.60, 1.19)	0.92 (0.68, 1.15)	1.18 (0.39, 1.96)
ASA score III-IV	1.47 (0.83, 2.10)	1.18 (0.86, 1.49)	1.18 (0.87, 1.49)	1.25 (0.39, 2.10)
Obesity (BMI≥30 kg/m^2)	0.97 (0.59, 1.36)	1.17 (0.79, 1.54)	1.22 (0.90, 1.55)	1.53 (0.52, 2.53)
Hypertension	0.96 (0.52, 1.41)	0.97 (0.61, 1.32)	0.97 (0.70, 1.28)	1.12 (0.24, 2.01)
Diabetes	1.78 (0.94, 2.63)	1.27 (0.87, 1.69)	1.33 (0.98, 1.68)	2.12 (0.63, 3.62)
Ischemic heart disease	0.89 (0.42, 1.36)	0.98 (0.58, 1.37)	0.85 (0.55, 1.08)	0.48 (0.06, 1.01)
Respiratory disease	0.89 (0.31, 1.46)	0.64 (0.24, 1.03)	0.75 (0.48, 1.02)	1.64 (0.35, 2.94)
**Surgical characteristics**				
Minimally invasive approach	0.73 (0.42, 1.05)	0.87 (0.58, 1.16)	0.86 (0.63, 1.09)	0.98 (0.29, 1.68)
Major hepatectomy	1.56 (0.92, 2.21)	1.14 (0.74, 1.54)	1.01 (0.73, 1.29)	0.60 (0.12, 1.08)
**Histological characteristics**				
Steatosis >33%	0.76 (0.37, 1.18)	0.82 (0.46, 1.17)	0.79 (0.55, 1.04)	1.57 (0.47, 2.67)
Cirrhosis	1.75 (0.97, 2.52)	1,33 (0.83, 1.84)	1.64 (1.19, 2.09) *	5.57 (1.72, 9.41) *
Multiple tumours	2.36 (1.29, 3.42) *	2.16 (1.36, 2.96) *	1.84 (1.26, 2.43) *	0.91 (0.01, 1.95)
Diameter >5cm	1.80 (1.05, 2.55) *	1.24 (0.84, 1.64)	1.17 (0.86, 1.48)	0.86 (0.27, 1.44)
Parenchymal R1	2.44 (1.07, 3.80) †	1.16 (0.44, 1.87)	1.67 (0.95, 2.41)	2.27 (0.19, 4.33)
Grade III-IV	1.47 (1.02, 1.92) *	1.11 (0.85, 1.38)	0.92 (0.52, 1.33)	0.76 (0.18, 1.34)
Macrovascular invasion	2.02 (1.37, 2.65) *	1.66 (1.05, 2.27) *	1.33 (0.89, 1.78)	0.56 (0.07, 1.06)
Microvascular invasion	3.08 (1.80, 4.36) *	2.01 (1.37, 2.64) *	1.46 (1.08, 1.84) *	0.57 (0.11, 1.02)

Hazard ratios for the 3-m, 18-m and 36-months were analyzed and not reported for the clarity of the table.

† refers to 3-month recurrence (Table 1 for details)

Table S2. Univariate analysis of time-specific hazard ratios for predictors of death without HCC recurrence.

Variable	6-m	12-m	24-m	60-m
**Clinical features**				
Age ≥70 years	0.97 (0.61,1.32)	1.05 (0.77, 1.32)	1.10 (0.79, 1.40)	1.10 (0.44, 1.76)
Male	0.99 (0.61,1.38)	1.06 (0.75, 1.37)	1.10 (0.78, 1.43)	1.22 (0.43, 2.03)
Inclusion period				
2001 – 2007	Ref.	Ref.	Ref.	Ref.
2008 – 2014	1.60 (0.71, 2.50)	0.87 (0.52, 1.22)	0.70 (0.43, 0.97)	0.82 (0.13, 1.50)
2015 – 2021	0.93 (0.60, 1.27)	1.04 (0.75, 1.34)	1.22 (0.89, 1.56)	2.28 (0.80, 3.77)
Geographic area				
Europe	Ref.	Ref.	Ref.	Ref.
North America	1.37 (0.87, 1.90)	0.94 (0.69, 1.19)	0.93 (0.67, 1.19)	2.18 (0.83, 3.54)
ASA score III-IV	1.45 (0.89, 2.01)	1.21 (0.88, 1.53)	1.20 (0.85, 1.53)	1.76 (0.66, 2.85)
Obesity (BMI≥30 kg/m^2)	0.96 (0.60, 1.32)	1.14 (0.84, 1.45)	1.32 (0.95, 1.68)	2.05 (0.84, 3.26)
Hypertension	0.99 (0.59, 1.41)	1.01 (0.70, 1.30)	1.02 (0.70, 1.34)	1.39 (0.38, 2.41)
Diabetes	1.89 (0.96, 2.83)	1.35 (0.99, 1.72)	1.36 (0.98, 1.73)	2.28 (0.90, 3.67)
Ischemic heart disease	0.95 (0.49, 1.40)	0.91 (0.60, 1.22)	0.86 (0.56, 1.16)	0.90 (0.23, 1.56)
Respiratory disease	0.63 (0.27, 1.01)	0.93 (0.27, 1.58)	0.77 (0.49, 1.05)	1.88 (0.66, 3.12)
**Surgical characteristics**				
Minimally invasive approach	0.58 (0.26, 0.87) †	0.80 (0.58, 1.03)	0.85 (0.61, 1.09)	1.21 (0.47, 1.96)
Major hepatectomy	1.68 (1.10, 2.27) *	1.10 (0.71, 1.48)	0.84 (0.49, 1.18)	0.77 (0.27, 1.26)
**Histological characteristics**				
Steatosis >33%	0.76 (0.48, 1.03)	0.79 (0.54, 1.03)	0.83 (0.55, 1.11)	1.04 (0.37, 1.70)
Cirrhosis	1.56 (0.96, 2.16)	1.32 (0.84, 1.79)	1.85 (1.38, 2.33) *	4.19 (1.74, 6.64) *
Multiple tumours	1.53(0.92, 2.14)	1.50 (0.81, 2.19)	1.39 (0.31, 2.42)	1.35 (0.21, 2.48)
Diameter >5cm	1.37 (0.98, 1.76)	1.25 (0.92, 1.59)	1.13 (0.82, 1.42)	1.03 (0.42, 1.65)
Parenchymal R1	1.59 (0.74, 2.45)	1.51 (0.76, 2.26)	1.56 (0.91, 2.22)	1.47 (0.66, 2.60)
Grade III-IV	1.22 (0.86, 1.58)	1.27 (0.91, 1.62)	1.23 (0.86, 1.59)	0.58 (0.13, 1.02)
Macrovascular invasion	1.45 (0.98, 1.92)	1.39 (0.92, 1.85)	1.28 (0.83, 1.72)	0.61 (0.05, 1.16)
Microvascular invasion	1.32 (0.98, 1.67)	1.26 (0.94, 1.57)	1.19 (0.90, 1.49)	0.60 (0.19, 1.03)

Hazard ratios for the 3-m, 18-m and 36-months were analyzed and not reported for the clarity of the table.
† refers to 3-month recurrence (Table 1 for details)
